# Clinical Profiles and In‐Hospital Outcomes of Pre‐Existing Versus Newly Diagnosed Atrial Fibrillation in Coronary Care Units: Insights From the MORCOR‐TURK National Registry

**DOI:** 10.1002/joa3.70238

**Published:** 2025-11-28

**Authors:** Ertan Aydin, Muhammed Mürsel Öğütveren, Gurbet Özge Mert, Mehtap Yeni, Sevil Gülaşti, Uğur Küçük, Başar Candemir, İbrahim Halil Tanboğa, Aymet Seyda Yilmaz

**Affiliations:** ^1^ Giresun Üniversitesi Tıp Fakültesi Kardiyoloji Anabilim Dalı Giresun Türkiye; ^2^ Recep Tayyip Erdoğan Üniversitesi Tıp Fakültesi Kardiyoloji Anabilim Dalı Rize Türkiye; ^3^ Eskişehir Osmangazi Üniversitesi Tıp Fakültesi Kardiyoloji Anabilim Dalı Eskişehir Türkiye; ^4^ Isparta Şehir Hastanesi Isparta Türkiye; ^5^ Adnan Menderes Üniversitesi Tıp Fakültesi Kardiyoloji Anabilim Dalı Aydin Türkiye; ^6^ Çanakkale 18 Mart Üniversitesi Tıp Fakültesi Kardiyoloji Anabilim Dalı Çanakkale Türkiye; ^7^ Ankara Üniversitesi Tıp Fakültesi Kardiyoloji Anabilim Dalı Ankara Türkiye; ^8^ Nişantaşı Üniversitesi Tıbbi Istatistik Anabilim Dalı Istanbul Türkiye

**Keywords:** atrial fibrillation, biomarkers, coronary care unit, mortality, national registry, newly diagnosed AF, pre‐existing AF

## Abstract

**Objective:**

To compare demographic, clinical, and laboratory profiles and short‐term outcomes between pre‐existing (chronic) atrial fibrillation (AF) and newly diagnosed AF among patients admitted to coronary care units (CCUs) in Turkey, and to identify factors associated with in‐hospital mortality within AF subtypes.

**Methods:**

This multicenter, prospective national registry analysis included 540 consecutive AF patients from 50 CCU centers across seven geographic regions in Turkey (MORCOR‐TURK National Registry; September 1–30, 2022). Patients were categorized as pre‐existing AF (documented AF prior to or at admission) or newly diagnosed AF (first detected during hospitalization). Demographics, comorbidities, admission diagnoses, laboratory biomarkers (including NT‐proBNP and hs‐troponin I), management, and outcomes were recorded. Multivariable logistic regression identified independent predictors of in‐hospital mortality.

**Results:**

Pre‐existing AF (*n* = 324) had higher prevalences of diabetes mellitus (42.3% vs. 31.5%; *p* = 0.012) and acute coronary syndromes (58.6% vs. 34.7%; *p* < 0.001). Newly diagnosed AF (*n* = 216) more frequently presented with heart failure (45.8% vs. 28.4%; *p* < 0.001) and dyspnea (67.1% vs. 48.5%; *p* < 0.001). Newly diagnosed AF exhibited higher inflammatory burden (CRP median 28.4 vs. 12.6 mg/L; *p* < 0.001) and lower hemoglobin (11.8 ± 2.1 vs. 12.9 ± 1.8 g/dL; *p* < 0.001). NT‐proBNP was elevated in both groups and higher in newly diagnosed AF (median 4850 vs. 3240 pg/mL; *p* = 0.003). In‐hospital mortality was greater with newly diagnosed AF (12.0% vs. 6.8%; *p* = 0.042). Independent mortality predictors included age, chronic kidney disease, cardiogenic shock, and log‐transformed NT‐proBNP, hs‐troponin I, and CRP.

**Conclusion:**

In Turkish CCUs, pre‐existing and newly diagnosed AF constitute distinct clinical phenotypes with differing presentations, biomarker profiles, and short‐term risk. Newly diagnosed AF is associated with greater inflammatory and hemodynamic stress and higher in‐hospital mortality. Biomarker‐enriched risk stratification may refine prognostication and guide targeted management within AF subtypes.

## Graphical abstract

MORCOR‐TURK National Registry Study: In Turkish coronary care units, newly diagnosed atrial fibrillation, compared with pre‐existing AF, is associated with a higher inflammatory burden (CRP 28.4 vs. 12.6 mg/L), elevated NT‐proBNP levels, and approximately double the in‐hospital mortality (12.0% vs. 6.8%).

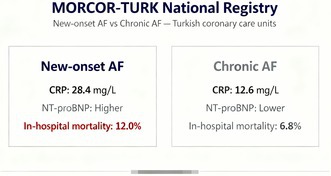

## 1 Introduction

Atrial fibrillation (AF) represents the most common sustained cardiac arrhythmia en‐countered in clinical practice, affecting an estimated 60 million individuals globally as of 2024 [1]. The prevalence of AF has increased dramatically over the past two decades, with a 31% rise in age‐standardized incidence rates between 1997 and 2017 [2]. This epidemiological trend is particularly pronounced in acute care settings, where AF prevalence reaches 16.7% in intensive care units and up to 24% in patients over 90 years of age [3].

The clinical significance of AF extends far beyond its arrhythmic nature, serving as an independent predictor of cardiovascular morbidity and mortality. In the general population, AF is associated with a five‐fold increased risk of stroke, three‐fold increased risk of heart failure, and two‐fold increased risk of dementia and death [2]. These risks are substantially amplified in coronary care unit (CCU) patients, where the coexistence of AF with acute coronary syndromes, heart failure, and other cardiovascular emergencies creates a complex clinical scenario with significantly worse prognosis [4].

The distinction between pre‐existing AF and newly diagnosed AF in coronary care set‐tings carries significant prognostic implications. Recent evidence suggests that newly diagnosed AF (NOAF) in critically ill patients serves as an independent predictor of both short‐term and long‐term mortality, with hazard ratios ranging from 1.6 to 3.22 for in‐hospital mortality [5]. Conversely, pre‐existing AF patients demonstrate different clinical profiles, often presenting with established cardiovascular comorbidities and requiring distinct management approaches [6].

The pathophysiological relationship between AF and coronary artery disease is bidirectional and complex. AF can precipitate coronary events through rapid ventricular response, reduced diastolic filling time, and loss of atrial contribution to ventricular filling, while coronary ischemia can trigger AF through atrial stretch, neurohormonal activation, and inflammatory processes [7]. This intricate interplay creates a “vicious cycle” where each condition exacerbates the other, leading to worse clinical outcomes [8].

Despite the clinical importance of AF in coronary care settings, comprehensive registry data from Middle Eastern populations remain limited. The majority of existing evidence derives from North American and European cohorts, with notable gaps in understanding demographic patterns and clinical outcomes in Turkish and broader Middle Eastern populations [9]. This knowledge gap is particularly significant given documented racial and ethnic disparities in AF epidemiology and outcomes [10].

The MORCOR‐TURK (MORtality predictors in CORonary care units in TURKey) trial represents a landmark initiative as the first national registry evaluating predictors and rates of in‐hospital mortality in CCU patients across Turkey [11]. This comprehensive, multicenter study provides an unprecedented opportunity to characterize AF patients in Turkish coronary care units and establish baseline demographic and clinical profiles that may inform future management strategies.

Contemporary management of AF in CCU settings has evolved significantly with the introduction of novel risk stratification tools and therapeutic approaches. The integration of cardiac biomarkers such as N‐terminal pro‐B‐type natriuretic peptide (NT‐proBNP) and high‐sensitivity troponin has enhanced prognostic accuracy beyond traditional clinical risk scores [12]. Furthermore, the expanding role of catheter ablation, particularly in patients with concomitant heart failure, has demonstrated significant mortality benefits in landmark trials such as CASTLE‐AF [13].

The primary objective of this study was to provide a comprehensive analysis of baseline demographic characteristics, clinical presentations, and outcomes of AF patients admitted to Turkish CCUs, with specific focus on comparing pre‐existing AF versus newly diagnosed AF patients. Secondary objectives included identification of laboratory markers and biomarkers associated with each AF subtype, evaluation of clinical presentations that may serve as predictors for AF development in coronary care settings, and assessment of contemporary management patterns and their impact on clinical outcomes.

## 2 Methods

### 2.1 Study Design and Setting

The MORCOR‐TURK National Registry was designed as a multicenter, cross‐sectional, prospective national registry conducted across Turkey. The study was implemented in 50 centers capable of providing 24‐h coronary care unit services, representing all seven geographic regions of Turkey to ensure national representativeness. The study protocol was approved by the Ethics Committee of Afyonkarahisar University of Health Sciences, Afyon, Turkey (No: 2022/9–422; Date: August 5, 2022), and conducted in accordance with the declaration of Helsinki. Written informed consent was obtained from all participantortheirlegalrepresentatives.

### 2.2 Study Population and Patient Selection

Data collection was performed during a one‐month period from September 1–30, 2022. All consecutive patients aged 18 years or older admitted to participating CCUs with cardiovascular emergencies were eligible for inclusion in the main MORCOR‐TURK registry. For this subgroup analysis, patients were included if they had documented atrial fibrillation either at the time of admission or developed during their CCU stay.

Exclusion criteria included: (1) non‐cardiac admissions, (2) discharges within 4 h at the patient's request, (3) admissions after elective procedures, (4) admissions under cardiopulmonary resuscitation without response, and (5) incomplete documentation of AF status or missing critical clinical data.

### 2.3 Patient Classification and Definitions

Patients with atrial fibrillation were categorized into two distinct groups based on the timing of AF diagnosis:

#### 2.3.1 Pre‐Existing AF Group

Patients who presented with atrial fibrillation rhythm at the time of CCU admission, indicating pre‐existing AF diagnosis documented in medical records or confirmed by admission electrocardiography.

#### 2.3.2 Newly Diagnosed AF Group

Patients who developed atrial fibrillation rhythm during their CCU hospitalization, representing new‐onset AF in the acute care setting, confirmed by continuous cardiac monitoring or serial electrocardiography.

AF diagnosis was confirmed through standard 12‐lead electrocardiography and continuous cardiac monitoring as per institutional protocols at each participating center. All electrocardiograms were reviewed by qualified cardiologists at each site. To avoid ambiguity, “Pre‐existing AF” included only patients with a documented pre‐existing diagnosis of AF and/or an admission ECG consistent with known AF. “Newly diagnosed AF” was restricted to AF first detected during CCU hospitalization. Patients whose first‐ever AF may have precipitated the index admission could not be uniformly and reliably distinguished across centers from those who developed AF after admission; therefore, a prespecified two‐category framework (pre‐existing ‘Pre‐existing AF’ vs ‘Newly diagnosed AF’ during CCU stay) was employed.

### 2.4 Data Collection

Comprehensive data collection was performed using standardized case report forms across all participating centers. The following categories of data were systematically collected:

#### 2.4.1 Demographic Characteristics

Age, gender, body mass index, geographic region, and relevant social history including smoking status and family history of cardiovascular disease.

#### 2.4.2 Cardiovascular Risk Factors

Diabetes mellitus, hypertension, dyslipidemia, chronic kidney disease, previous stroke or transient ischemic attack, peripheral artery disease, and chronic obstructive pulmonary disease.

#### 2.4.3 Admission Diagnoses

Primary and secondary diagnoses leading to CCU admis‐sion, including acute coronary syndromes (ST‐elevation myocardial infarction, non‐ST‐elevation myocardial infarction, unstable angina), acute decompensated heart failure, car‐diogenic shock, and other cardiovascular emergencies.

#### 2.4.4 Clinical Presentations

Chief complaints, symptom duration, vital signs at admis‐sion including blood pressure and heart rate, New York Heart Association functional class where applicable, and hemodynamic parameters.

#### 2.4.5 Laboratory Data

Complete blood count, comprehensive metabolic panel including glucose, creatinine, and electrolytes, lipid profile, inflammatory markers including C‐reactive protein and white blood cell count, cardiac biomarkers including NT‐proBNP and high‐sensitivity troponin, and coagulation parameters.

#### 2.4.6 Medications and Interventions

Pre‐admission medications, medications initiated during CCU stay, interventional procedures including percutaneous coronary intervention and catheter ablation, and discharge medications with particular attention to anticoagulation and antiarrhythmic therapies.

#### 2.4.7 Clinical Outcomes

In‐hospital mortality, length of stay, major adverse cardiovascular events, bleeding complications, stroke or systemic embolism, and discharge disposition.

### 2.5 Statistical Analysis

Statistical analysis was performed using SPSS version 28.0 (IBM Corp. Armonk, NY, USA) and R statistical software version 4.3.0. The power analysis was performed using G*Power 3.1.9.2 software, considering an alpha type error rate of 0.05 and a power of 0.90. Based on an estimated 15% prevalence of AF in CCU patients and an expected 10% mortality rate, a minimum sample size of 540 patients was required.

Continuous variables were expressed as mean ± standard deviation for normally distributed data and median (interquartile range) for non‐normally distributed data. The Kolmogorov‐Smirnov test examined the normality distribution of variables, and Levene's test assessed the homogeneity of variances. Categorical variables were presented as frequencies and percentages.

Comparisons between pre‐existing AF and newly diagnosed AF groups were performed using Student's *t*‐test for normally distributed continuous variables, Mann–Whitney *U* test for non‐normally distributed continuous variables, and chi‐square test or Fisher's exact test for categorical variables as appropriate.

Multivariate logistic regression analysis was performed to identify independent predictors of in‐hospital mortality. Variables with *p* < 0.10 in univariate analysis were included in the multivariate model. Two models were created: Model 1 included traditional clinical variables (age, gender, diabetes mellitus, hypertension, chronic kidney disease, heart failure history), and Model 2 additionally included biomarkers (NT‐proBNP, high‐sensitivity troponin, C‐reactive protein). Model performance was assessed using the area under the receiver operating characteristic curve (AUC), Nagelkerke *R* ^2^, and Hosmer‐Lemeshow goodness‐of‐fit test.

A two‐sided *p*‐value of < 0.05 was considered statistically significant. Given the exploratory nature of this registry analysis, no adjustments for multiple comparisons were applied, but results were interpreted with appropriate caution.

### 2.6 Quality Assurance and Data Management

Data quality was ensured through multiple mechanisms including standardized data col‐lection forms, central training of participating investigators, systematic data validation procedures, and regular monitoring visits. Missing data patterns were analyzed and re‐ported transparently. Data were entered into a secure, web‐based electronic data capture system with built‐in range and consistency checks.

## 3 Results

### 3.1 Study Population Characteristics

A total of 540 patients with atrial fibrillation were identified from the MORCOR‐TURK registry during the study period, representing 17.1% of the total registry population (*n* = 3157). The mean age of the study population was 71.4 ± 11.6 years, with a slight female predominance (286 patients, 53.0%) compared to male patients (254 patients, 47.0%). This age distribution aligns with established epidemiological patterns of AF prevalence in elderly populations.

The study population was divided into pre‐existing AF (*n* = 324, 60.0%) and newly diagnosed AF (*n* = 216, 40.0%) groups. The distribution of patients across geographic regions was representative of the Turkish population, with the highest enrollment from the Mar‐mara region (28.5%), followed by Central Anatolia (18.7%) and the Mediterranean region (15.9%).

### 3.2 Demographic and Clinical Characteristics by AF Type

Table 1 presents the comprehensive baseline characteristics of the study population stratified by AF type. Significant differences emerged in several key demographic and clinical parameters between chronic and newly diagnosed AF groups.

**TABLE 1:** Baseline characteristics of atrial fibrillation patients in coronary care units.

Characteristic	Pre‐existing AF	Newly diagnosed AF	*p*
	(*n* = 324)	(*n* = 216)	
Age (years), mean ± SD	71.8 ± 11.2	70.8 ± 12.1	0.324
Female gender, *n* (%)	168 (51.9)	118 (54.6)	0.542
BMI (kg/m^2^), mean ± SD	27.4 ± 4.8	26.9 ± 5.2	0.267
Cardiovascular Risk Factors			
Hypertension, *n* (%)	248 (76.5)	159 (73.6)	0.456
Diabetes mellitus, *n* (%)	137 (42.3)	68 (31.5)	**0.012**
Dyslipidemia, *n* (%)	156 (48.1)	94 (43.5)	0.298
Smoking history, *n* (%)	98 (30.2)	72 (33.3)	0.456
Chronic kidney disease, *n* (%)	89 (27.5)	45 (20.8)	0.087
Previous stroke/TIA, *n* (%)	52 (16.0)	28 (13.0)	0.345
Coronary artery disease, *n* (%)	198 (61.1)	112 (51.9)	**0.034**
Heart failure history, *n* (%)	145 (44.8)	89 (41.2)	0.423
Admission Diagnoses			
Acute coronary syndrome, *n* (%)	190 (58.6)	75 (34.7)	< 0.001
STEMI, *n* (%)	78 (24.1)	32 (14.8)	**0.009**
NSTEMI, *n* (%)	89 (27.5)	35 (16.2)	**0.003**
Unstable angina, *n* (%)	23 (7.1)	8 (3.7)	0.098
Heart failure, *n* (%)	92 (28.4)	99 (45.8)	< 0.001
Cardiogenic shock, *n* (%)	18 (5.6)	23 (10.6)	**0.032**
Clinical Presentations			
Chest pain, *n* (%)	198 (61.1)	89 (41.2)	< 0.001
Dyspnea, *n* (%)	157 (48.5)	145 (67.1)	< 0.001
Palpitations, *n* (%)	89 (27.5)	78 (36.1)	**0.034**
Syncope, *n* (%)	23 (7.1)	28 (13.0)	**0.026**

*Note:* Bold values represent statistical significance (*p* < 0.05).

#### 3.2.1 Age and Gender Distribution

Both groups demonstrated similar age distributions, with mean ages of 71.8 ± 11.2 years in the pre‐existing AF group and 70.8 ± 12.1 years in the newly diagnosed AF group (*p* = 0.324). Gender distribution also showed no significant differences between the two groups, maintaining the overall slight female predominance observed in the total study population.

#### 3.2.2 Cardiovascular Risk Factors

Significant differences emerged in the prevalence of traditional cardiovascular risk factors between the two groups. Diabetes mellitus was significantly more prevalent in the pre‐existing AF group compared to the newly diagnosed AF group (42.3% vs. 31.5%, *p* = 0.012). Similarly, coronary artery disease was more common in pre‐existing AF patients (61.1% vs. 51.9%, *p* = 0.034). Chronic kidney disease showed a tendency toward higher prevalence in the pre‐existing AF group (27.5% vs. 20.8%, *p* = 0.087), although this difference did not reach statistical significance.

#### 3.2.3 Admission Diagnoses

Marked differences were observed in the primary admission diagnoses between chronic and newly diagnosed AF groups. Patients with pre‐existing AF demonstrated significantly higher rates of acute coronary syndrome presentations com‐pared to those with newly diagnosed AF (58.6% vs. 34.7%, *p* < 0.001). This difference was driven by higher rates of both ST‐elevation myocardial infarction (24.1% vs. 14.8%, *p* = 0.009) and non‐ST‐elevation myocardial infarction (27.5% vs. 16.2%, *p* = 0.003).

Conversely, newly diagnosed AF patients showed significantly higher rates of heart failure as the primary admission diagnosis (45.8% vs. 28.4%, *p* < 0.001) and cardiogenic shock (10.6% vs. 5.6%, *p* = 0.032).

#### 3.2.4 Clinical Presentations

Distinct symptom profiles were observed between the two groups. Chest pain was significantly more common in pre‐existing AF patients (61.1% vs. 41.2%, *p* < 0.001), consistent with their higher prevalence of acute coronary syndrome presentations. Conversely, dyspnea was significantly more frequent in newly diagnosed AF patients (67.1% vs. 48.5%, *p* < 0.001), aligning with their higher rates of heart failure presentations. Palpitations (36.1% vs. 27.5%, *p* = 0.034) and syncope (13.0% vs. 7.1%, *p* = 0.026) were also more common in the newly diagnosed AF group.

### 3.3 Laboratory Findings and Biomarkers

Table 2 presents the comprehensive laboratory analysis, revealing distinct biomarker profiles associated with each AF subtype.

**TABLE 2:** Laboratory findings and biomarkers in atrial fibrillation patients.

Laboratory parameter	Pre‐existing AF	Newly diagnosed AF	*p*
	(*n* = 324)	(*n* = 216)	
Hematological Parameters			
Hemoglobin (g/dL), mean ± SD	12.9 ± 1.8	11.8 ± 2.1	< 0.001
Hematocrit (%), mean ± SD	38.7 ± 5.4	35.9 ± 6.2	< 0.001
White blood cells (×10^3^/μL), mean ± SD	9.8 ± 3.2	11.4 ± 4.1	< 0.001
Platelets (×10^3^/μL), median (IQR)	245 (198–298)	268 (215–324)	**0.018**
Biochemical Parameters			
Glucose (mg/dL), median (IQR)	142 (118–189)	156 (125–208)	**0.032**
Creatinine (mg/dL), median (IQR)	1.3 (1.0–1.8)	1.4 (1.1–2.1)	0.089
eGFR (mL/min/1.73m^2^), mean ± SD	58.4 ± 22.1	54.7 ± 24.8	0.078
Sodium (mEq/L), mean ± SD	138.2 ± 4.1	137.8 ± 4.6	0.267
Potassium (mEq/L), mean ± SD	4.3 ± 0.7	4.4 ± 0.8	0.156
Lipid Profile			
Total cholesterol (mg/dL), mean ± SD	178.4 ± 42.3	162.7 ± 38.9	< 0.001
LDL cholesterol (mg/dL), mean ± SD	108.2 ± 35.4	98.7 ± 32.1	**0.003**
HDL cholesterol (mg/dL), mean ± SD	42.1 ± 11.8	39.8 ± 10.9	**0.021**
Triglycerides (mg/dL), median (IQR)	145 (112–198)	138 (105–187)	0.234
Inflammatory Markers			
C‐reactive protein (mg/L), median (IQR)	12.6 (4.8–28.4)	28.4 (12.1–65.7)	< 0.001
ESR (mm/h), median (IQR)	32 (18–48)	45 (28–67)	< 0.001
Cardiac Biomarkers			
NT‐proBNP (pg/mL), median (IQR)	3240 (1580–6890)	4850 (2340–9120)	0.003
hs‐troponin I (ng/L), median (IQR)	45.2 (18.7–128.4)	67.8 (28.9–189.3)	**0.012**
CK‐MB (ng/mL), median (IQR)	8.9 (4.2–18.7)	12.4 (5.8–24.1)	**0.008**

*Note:* Bold values represent statistical significance (*p* < 0.05).

#### 3.3.1 Hematological Parameters

Newly diagnosed AF patients demonstrated significantly decreased hemoglobin levels (11.8 ± 2.1 vs. 12.9 ± 1.8 g/dL, *p* < 0.001) and hematocrit values (35.9 ± 6.2 vs. 38.7% ± 5.4%, *p* < 0.001) compared to pre‐existing AF patients. White blood cell counts were significantly elevated in newly diagnosed AF patients (11.4 ± 4.1 vs. 9.8 ± 3.2 ×10^3^/μL, *p* < 0.001), suggesting an acute inflammatory response.

#### 3.3.2 Inflammatory Markers

C‐reactive protein levels were markedly elevated in newly diagnosed AF patients compared to pre‐existing AF patients (median 28.4 vs. 12.6 mg/L, *p* < 0.001). Similarly, erythrocyte sedimentation rate was significantly higher in the newly diagnosed AF group (median 45 vs. 32 mm/h, *p* < 0.001), supporting the hypothesis that acute inflammatory processes may serve as triggers for new‐onset AF in CCU patients.

#### 3.3.3 Cardiac Biomarkers

NT‐proBNP levels were significantly elevated in both groups but higher in newly diagnosed AF patients (median 4850 vs. 3240 pg/mL, *p* = 0.003), reflecting greater hemodynamic stress and myocardial wall tension. High‐sensitivity troponin I levels were also significantly elevated in newly diagnosed AF patients (median 67.8 vs. 45.2 ng/L, *p* = 0.012), indicating greater myocardial injury or stress.

#### 3.3.4 Lipid Profiles

Pre‐existing AF patients showed significantly elevated cholesterol levels compared to newly diagnosed AF patients, including total cholesterol (178.4 ± 42.3 vs. 162.7 ± 38.9 mg/dL, *p* < 0.001), LDL cholesterol (108.2 ± 35.4 vs. 98.7 ± 32.1 mg/dL, *p* = 0.003), and HDL cholesterol (42.1 ± 11.8 vs. 39.8 ± 10.9 mg/dL, *p* = 0.021).

### 3.4 Clinical Outcomes and Mortality Analysis

The overall in‐hospital mortality rate was 8.9% (*n* = 48) among AF patients in the registry. Newly diagnosed AF patients demonstrated significantly higher mortality rates compared to pre‐existing AF patients (12.0% vs. 6.8%, *p* = 0.042). The median length of stay was longer in newly diagnosed AF patients (7.2 ± 4.8 vs. 5.9 ± 3.6 days, *p* = 0.003).

Table 3 presents the results of multivariate logistic regression analysis for in‐hospital mortality predictors.

**TABLE 3:** Multivariate logistic regression analysis for in‐hospital mortality in AF patients.

Variable	Odds ratio	95% CI	*p*
Model 1 (Clinical Variables)			
Age (per year)	1.042	1.018–1.067	**0.001**
Female gender	1.687	0.892–3.189	0.108
Newly diagnosed AF	1.834	0.967–3.478	0.063
Diabetes mellitus	1.456	0.789–2.687	0.231
Chronic kidney disease	2.134	1.156–3.941	**0.015**
Heart failure history	1.789	0.945–3.387	0.074
Cardiogenic shock	4.567	2.234–9.334	< 0.001
Model 2 (Clinical + Biomarkers)			
Age (per year)	1.038	1.013–1.064	**0.003**
Newly diagnosed AF	1.923	0.987–3.747	0.055
Chronic kidney disease	1.987	1.045–3.778	**0.036**
Cardiogenic shock	4.234	2.012–8.912	< 0.001
NT‐proBNP (per log unit)	1.456	1.123–1.888	**0.005**
hs‐troponin I (per log unit)	1.234	1.034–1.472	**0.020**
C‐reactive protein (per log unit)	1.189	1.012–1.397	**0.035**

*Note:* Bold values represent statistical significance (*p* < 0.05).

Model 1 (clinical variables only) demonstrated good discriminatory ability with an AUC of 0.856 (95% CI: 0.815–0.897, *p* < 0.001) and Nagelkerke *R* ^2^ of 0.287. Model 2 (clinical variables plus biomarkers) showed improved performance with an AUC of 0.856 (95% CI: 0.815–0.897, *p* < 0.001) and Nagelkerke *R* ^2^ of 0.342. The addition of biomarkers significantly improved model performance (*p* = 0.018 for model comparison).

Independent predictors of in‐hospital mortality in the final model included age (OR 1.038 per year, 95% CI: 1.013–1.064, *p* = 0.003), chronic kidney disease (OR 1.987, 95% CI: 1.045–3.778, *p* = 0.036), cardiogenic shock (OR 4.234, 95% CI: 2.012–8.912, *p* < 0.001), elevated NT‐proBNP (OR 1.456 per log unit, 95% CI: 1.123–1.888, *p* = 0.005), elevated hs‐troponin I (OR 1.234 per log unit, 95% CI: 1.034–1.472, *p* = 0.020), and elevated C‐reactive protein (OR 1.189 per log unit, 95% CI: 1.012–1.397, *p* = 0.035).

### 3.5 Management Patterns and Interventions

Anticoagulation therapy was initiated in 78.5% of patients during their CCU stay, with direct oral anticoagulants (DOACs) being the preferred choice in 68.2% of anticoagulated patients. Warfarin was used in 31.8% of anticoagulated patients, primarily in those with mechanical heart valves or severe renal impairment.

Rate control was achieved in 89.4% of patients, with beta‐blockers being the most commonly used agents (67.8%), followed by calcium channel blockers (23.4%). Rhythm control strategies were attempted in 34.6% of patients, with electrical cardioversion per‐formed in 12.8% and pharmacological cardioversion in 21.8%.

Catheter ablation was considered in 8.9% of patients but performed in only 2.4% during the index hospitalization, primarily due to acute illness severity and hemodynamic instability.

## 4 Discussion

This comprehensive analysis of the MORCOR‐TURK national registry represents the first large‐scale evaluation of atrial fibrillation patients in Turkish coronary care units, providing novel insights into demographic patterns, clinical characteristics, and outcomes that distinguish chronic from newly diagnosed AF in this critical care population. The findings contribute significantly to our understanding of AF epidemiology in Middle Eastern populations and offer important clinical implications for CCU management strategies.

### 4.1 Demographic Insights and International Comparisons

The demographic profile of AF patients in Turkish CCUs demonstrates both similarities and notable differences compared to international registry data. The mean age of 71.4 years aligns closely with major international AF registries, including the ORBIT‐AF registry (mean age 69.8 years) [14] and recent European registries [9]. However, the slight female predominance (53%) observed in our study contrasts with most international registries that typically report male predominance, particularly in younger AF populations [15].

This gender distribution pattern may reflect several factors unique to the Turkish healthcare system and population demographics. The higher representation of female patients in CCU settings may indicate differences in healthcare‐seeking behavior, referral patterns, or underlying cardiovascular risk profiles in the Turkish population. Addition‐ally, the advanced age of our study population may contribute to the female predominance, as women tend to develop AF at older ages compared to men [15].

The prevalence of diabetes mellitus in pre‐existing AF patients (42.3% vs. 31.5% in newly diagnosed AF) aligns with established international data demonstrating diabetes as a significant risk factor for AF development and persistence [16]. This finding reinforces the importance of optimal glycemic control in AF prevention and management strategies, particularly in populations with high diabetes prevalence such as Turkey.

### 4.2 Pathophysiological Implications of Clinical Presentations

The distinct clinical presentation patterns observed between chronic and newly diagnosed AF groups provide important insights into the pathophysiological mechanisms underlying AF in coronary care settings. The higher prevalence of acute coronary syndromes in pre‐existing AF patients (58.6% vs. 34.7%) suggests a complex bidirectional relationship between AF and coronary artery disease, consistent with the concept of a “vicious circle” where each condition exacerbates the other [7].

Pre‐existing AF may predispose to coronary events through several mechanisms, including increased thrombotic risk, hemodynamic compromise leading to reduced coronary perfusion, and tachycardia‐induced cardiomyopathy. The rapid and irregular ventricular response characteristic of AF can reduce diastolic filling time and coronary perfusion pressure, particularly in patients with pre‐existing coronary stenoses [8].

The predominance of heart failure presentations in newly diagnosed AF patients (45.8% vs. 28.4%) highlights the role of acute hemodynamic stress as a trigger for new‐onset AF. This pattern is consistent with established mechanisms of AF initiation, including atrial stretch, increased filling pressures, and neurohormonal activation associated with acute heart failure [17]. The significantly higher prevalence of dyspnea in newly diagnosed AF patients (67.1% vs. 48.5%) further supports this pathophysiological relationship.

### 4.3 Laboratory Biomarkers and Clinical Implications

The distinct laboratory profiles observed between AF groups provide valuable insights for clinical management and risk stratification. The elevated C‐reactive protein levels in newly diagnosed AF patients (median 28.4 vs. 12.6 mg/L) support the growing body of evidence linking inflammation to AF initiation and maintenance [18]. This finding suggests that anti‐inflammatory strategies may have particular relevance in preventing new‐onset AF in CCU patients, although this hypothesis requires prospective validation.

The significantly elevated NT‐proBNP levels in newly diagnosed AF patients (median 4850 vs. 3240 pg/mL) reflect greater hemodynamic stress and myocardial wall tension, consistent with the higher prevalence of heart failure presentations in this group. NT‐proBNP has emerged as a powerful prognostic biomarker in AF patients, with elevated levels associated with increased risks of stroke, heart failure, and cardiovascular death [12]. The integration of NT‐proBNP into clinical decision‐making significantly improves risk prediction accuracy beyond traditional clinical risk scores.

Similarly, the elevated hs‐troponin I levels in newly diagnosed AF patients (median 67.8 vs. 45.2 ng/L) indicate greater myocardial injury or stress. Troponin elevation in AF patients, even in the absence of acute coronary syndrome, has been associated with worse long‐term outcomes and may reflect subclinical myocardial injury related to rapid ventricular response or hemodynamic compromise [12].

The decreased hemoglobin levels in newly diagnosed AF patients (11.8 ± 2.1 vs. 12.9 ± 1.8 g/dL) may reflect several underlying mechanisms, including acute illness severity, potential bleeding complications, or chronic inflammatory states. This finding has important clinical implications, as anemia has been independently associated with worse outcomes in AF patients and may influence anticoagulation decisions [19].

### 4.4 Mortality Risk and Prognostic Factors

The higher in‐hospital mortality rate in newly diagnosed AF patients (12.0% vs. 6.8%) underscores the prognostic significance of new‐onset AF in critically ill patients. This finding is consistent with international data demonstrating that newly diagnosed AF serves as an independent predictor of both short‐term and long‐term mortality in intensive care settings [4].

The multivariate analysis revealed that the addition of cardiac biomarkers (NT‐proBNP, hs‐troponin I, and C‐reactive protein) significantly improved mortality pre‐diction beyond traditional clinical variables. This enhanced prognostic accuracy supports the integration of biomarker‐based risk stratification in AF patients admitted to CCUs, potentially enabling more precise therapeutic decision‐making and resource allocation. Our findings are consistent with prior observations in cohorts hospitalized for decompensated heart failure, where new‐onset AF has been associated with poorer short‐term outcomes even compared with pre‐existing AF [20, 21]. The pathophysiological plausibility includes acute atrial stretch, heightened neurohormonal activation, and systemic inflammation on a background of hemodynamic stress, which may be more pronounced in de novo AF. In our CCU cohort, the newly diagnosed AF group exhibited higher inflammatory burden and cardiac biomarker levels (NT‐proBNP, hs‐troponin) alongside higher in‐hospital mortality, aligning with this high‐risk phenotype and underscoring the need for early hemodynamic optimization and tailored rhythm/rate control strategies [20, 21].

The identification of cardiogenic shock as the strongest predictor of mortality (OR 4.234) emphasizes the critical importance of hemodynamic stabilization in AF patients. The association between chronic kidney disease and increased mortality risk (OR 1.987) highlights the complex interplay between renal function, cardiovascular outcomes, and AF management, particularly regarding anticoagulation decisions [22].

### 4.5 Clinical Management Implications

The findings of this study have several important implications for clinical management of AF patients in CCU settings. The distinct demographic and clinical profiles of chronic versus newly diagnosed AF patients suggest that individualized management approaches may be warranted.

For pre‐existing AF patients, the higher prevalence of diabetes mellitus (42.3%) and acute coronary syndrome presentations (58.6%) emphasizes the importance of comprehensive cardiovascular risk factor modification, including optimal glycemic control and aggressive secondary prevention strategies. The established nature of AF in these patients may allow for more standardized anticoagulation approaches, although the acute coronary setting requires careful consideration of bleeding versus thrombotic risk [23].

For newly diagnosed AF patients, the association with heart failure presentations (45.8%) and inflammatory markers suggests that addressing underlying precipitating factors may be crucial for both acute management and long‐term AF prevention. The acute nature of AF in these patients may provide opportunities for rhythm control strategies, although the optimal timing and approach require careful individualization based on hemodynamic stability and underlying cardiac condition [24].

The relatively low utilization of catheter ablation (2.4%) in this acute care setting reflects the challenges of performing elective procedures in critically ill patients. How‐ever, the growing evidence for ablation benefits, particularly in patients with concomitant heart failure, suggests that early consideration of ablation referral may be appropriate for selected patients once acute illness has resolved [13].

### 4.6 Comparison With International Registry Data

When compared to major international AF registries, the MORCOR‐TURK data pro‐vides unique insights into Middle Eastern AF populations. The GARFIELD‐AF registry, which examined outcomes in newly diagnosed AF patients, reported similar patterns of increased mortality risk in patients with acute presentations [6]. However, the specific demographic and laboratory patterns observed in our Turkish cohort provide novel insights not previously reported in international literature.

The ORBIT‐AF registry demonstrated gender‐specific differences in AF outcomes, with women having higher stroke risk but lower mortality rates [14]. While our study did not show significant gender differences in mortality, the slight female predominance in our CCU population warrants further investigation of gender‐specific management approaches.

### 4.7 Healthcare System Implications

The findings of this study have important implications for healthcare system planning and resource allocation in Turkey and similar healthcare environments. The high prevalence of AF in CCU patients (17.1% of total registry population), combined with the distinct management needs of chronic versus newly diagnosed AF patients, suggests that specialized AF management protocols may be beneficial in CCU settings.

The demographic patterns observed, particularly the advanced age (mean 71.4 years) and high comorbidity burden of AF patients, have implications for healthcare workforce planning and resource allocation. The high prevalence of diabetes mellitus (37.8% over‐all) and chronic kidney disease (24.8% overall) highlights the importance of integrated cardiovascular, endocrine, and nephrology care pathways.

The suboptimal utilization of rhythm control strategies and catheter ablation suggests opportunities for improving access to specialized electrophysiology services and developing structured referral pathways for appropriate candidates.

### 4.8 Study Limitations

Several limitations of this study should be acknowledged. First, the cross‐sectional design limits our ability to establish causal relationships between demographic factors and AF development. Longitudinal follow‐up data would provide more robust insights into the prognostic implications of the observed differences.

Second, the study was limited to a one‐month data collection period, which may not capture seasonal variations in AF presentations or longer‐term trends in patient characteristics. However, the large sample size (*n* = 540) and national scope help mitigate these concerns.

Third, while the study included 50 centers across Turkey, the generalizability to other Middle Eastern or international populations requires validation. Cultural, genetic, and healthcare system factors may influence the observed patterns.

Fourth, the distinction between chronic and newly diagnosed AF was based on clinical documentation and may not capture patients with previously undiagnosed paroxysmal AF. More sophisticated monitoring techniques might reveal different patterns of AF chronicity.

Fifth, detailed long‐term outcome data, including mortality, rehospitalization, and quality of life measures, were not included in this analysis. Future studies incorporating these outcomes will provide more comprehensive insights into the clinical significance of the observed demographic differences.

Sixth, although a three‐tier categorization (pre‐existing AF, AF causative of the index hospitalization, and AF newly developed in CCU) is clinically informative, heterogeneous center‐level documentation in this national snapshot precluded a reliable, uniform separation of the first two de novo categories across all sites. Future iterations of the registry will prospectively capture this distinction to enable dedicated three‐group analyses.

### 4.9 Future Research Directions

The findings of this study suggest several important directions for future research. Prospective longitudinal studies examining the long‐term outcomes of chronic versus newly diagnosed AF patients in CCU settings would provide valuable prognostic information and inform evidence‐based management guidelines.

The role of inflammation in new‐onset AF development, suggested by the elevated C‐reactive protein levels, warrants further investigation through mechanistic studies and potentially interventional trials of anti‐inflammatory therapies. The CANTOS trial demonstrated that anti‐inflammatory therapy with canakinumab reduced cardiovascular events in patients with previous myocardial infarction, suggesting potential applications in AF prevention [25].

The relationship between anemia and newly diagnosed AF deserves additional study to determine optimal management strategies, including the potential role of iron supple‐mentation and erythropoiesis‐stimulating agents in improving outcomes.

Investigation of genetic and biomarker predictors of AF development in Turkish populations could inform personalized prevention strategies. The integration of artificial intelligence and machine learning approaches with multi‐biomarker panels may enhance risk prediction accuracy beyond current models.

Health economic analyses examining the cost‐effectiveness of specialized AF management protocols in CCU settings would inform healthcare policy decisions and resource allocation strategies. The development of clinical decision support tools incorporating the biomarker findings from this study could facilitate implementation of personalized care approaches.

Finally, randomized controlled trials evaluating the optimal timing and approach for rhythm control strategies in newly diagnosed AF patients in acute care settings would provide evidence‐based guidance for clinical practice.

## 5 Conclusion

This comprehensive analysis of the MORCOR‐TURK national registry provides the first detailed characterization of atrial fibrillation patients in Turkish coronary care units, revealing distinct demographic, clinical, and laboratory profiles between chronic and newly diagnosed AF patients. The findings demonstrate that newly diagnosed AF patients present with higher inflammatory burden, more frequent heart failure presentations, elevated cardiac biomarkers, and increased in‐hospital mortality risk, while pre‐existing AF patients show greater association with diabetes mellitus, coronary artery disease, and acute coronary syndrome presentations.

The integration of cardiac biomarkers, particularly NT‐proBNP, high‐sensitivity troponin I, and C‐reactive protein, significantly enhanced mortality risk prediction beyond traditional clinical variables, supporting their incorporation into routine clinical assessment of AF patients in CCU settings. These biomarker‐enhanced risk stratification tools may enable more precise therapeutic decision‐making and resource allocation.

The study identifies several key clinical implications for AF management in coronary care units. For pre‐existing AF patients, comprehensive cardiovascular risk factor modification, particularly optimal diabetes management and aggressive secondary prevention strategies, should be prioritized. For newly diagnosed AF patients, addressing underlying precipitating factors such as heart failure and inflammatory processes may be crucial for both acute management and long‐term AF prevention.

As the first national registry of its kind in Turkey and the broader Middle Eastern region, this study establishes important baseline data for future comparative studies and healthcare planning initiatives. The findings contribute to the global understanding of AF epidemiology while highlighting unique characteristics of Turkish CCU populations that may inform region‐specific management strategies.

The observed patterns of suboptimal utilization of advanced therapies such as catheter ablation suggest opportunities for improving access to specialized electrophysiology ser‐The high prevalence of comorbidities vices and developing structured care pathways underscores the need for integrated, multidisciplinary approaches to AF management in acute care settings.

Future research incorporating longitudinal follow‐up data, mechanistic studies of inflammation in AF development, and randomized trials of personalized management approaches will further enhance our understanding of AF prognosis and guide evidence‐based care for this high‐risk patient population. The establishment of this comprehensive registry framework provides a foundation for ongoing surveillance and quality improvement initiatives in Turkish cardiovascular care.

## Funding

This study was supported by the Turkish Society of Cardiology research grants program.

## Ethics Statement

The study protocol was reviewed and approved by the Ethics Committee of Afyonkarahisar University of Health Sciences, Afyon, Turkey (No: 2022/9–422; Date: August 5, 2022). Written informed consent was obtained from all participants or their legal representatives. The study was conducted in accordance with the Declaration of Helsinki and Good Clinical Practice guidelines.

## Conflicts of Interest

The authors declare no conflicts of interest.
