# Correction to “Clinical Profiles and In‐Hospital Outcomes of Pre‐Existing Versus Newly Diagnosed Atrial Fibrillation in Coronary Care Units: Insights From the MORCOR‐TURK National Registry”

**DOI:** 10.1002/joa3.70260

**Published:** 2025-12-30

E. Aydin, M. M. Öğütveren, G. Ö. Mert, et al., “Clinical Profiles and In‐Hospital Outcomes of Pre‐Existing Versus Newly Diagnosed Atrial Fibrillation in Coronary Care Units: Insights From the MORCOR‐TURK National Registry,” *Journal of Arrhythmia* 41, no. 6 (2025): e70238, https://doi.org/10.1002/joa3.70238.

The name of the last author is corrected from “Aymet Seyda Yilmaz” to “Ahmet Seyda Yılmaz.” The online version of this article has been corrected accordingly.

We apologize for this error.

